# New players and targets in mannose 6-phosphate-dependent lysosomal sorting

**DOI:** 10.1038/s44318-024-00310-2

**Published:** 2024-11-25

**Authors:** Nikita Zubkov, Sean Munro

**Affiliations:** MRC Laboratory of Molecular Biology, Cambridge, CB2 0QH UK

**Keywords:** Membranes & Trafficking, Organelles, Post-translational Modifications & Proteolysis

## Abstract

A new study shows how a Golgi-resident protein is sent to the lysosome for degradation.

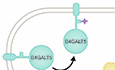

## Teaser

The Golgi apparatus serves multiple crucial roles in the cell. These roles include the addition and subsequent modification of glycans, not only to newly-made proteins arriving from the endoplasmic reticulum, but also to precursors during the biosynthesis of glycolipids. New research by Brauer et al, (2024) now provides a conceptual framework that connects the use of glycosylation as a protein targeting signal with its use as a marker for Golgi quality control.

Amidst the wide variety of glycan modifications that are carried out in the Golgi apparatus, the addition of mannose 6-phosphate (M6P) to the N-linked glycans of lysosome-resident hydrolase enzymes stands out. These M6P-modified glycans are recognised by mannose 6-phosphate receptors that cycle between the Golgi and endosomes, diverting lysosomal hydrolases from the secretory pathway to their correct lysosomal destination. This system delivers more than 60 different enzymes to the lysosome, thus supplying this major degradative organelle with the complete set of enzymes it needs to function. In humans, the deficiency of even one of these enzymes can result in a fatal lysosomal storage disorder (Braulke et al, 2024). The enzyme that catalyses the addition of M6P-tags is a membrane-spanning cis-Golgi protein called *N*-acetylglucosamine-1-phosphotransferase (GNPT). GNPT is comprised of three subunits: two of these subunits are generated after the cleavage of a single gene product (GNPTAB). Recent studies have shown that the correct localisation of GNPT requires a small membrane protein LYSET, but the mechanism that mediates GNPT’s localisation has remained unclear (Pechincha et al, 2024; Richards et al, 2024).

Rather like standing still by walking against the direction of an escalator, if Golgi-resident enzymes are to be correctly localised within the maturing Golgi cisternae, they must be continuously trafficked in a retrograde direction within COPI-coated vesicles as the Golgi cisternae mature through the stack. The retention of a vast number of diverse Golgi residents is managed in this way as they are recognised by COPI adaptors such as the cytosolic protein GOLPH3 or its closely related paralog GOLPH3L (Welch et al, 2021; Rizzo et al, 2021). As a complicating factor, the catalytic domains of some Golgi-resident enzymes are released into the lumen and secreted after being cleaved by an intramembrane protease, SPPL3 (Truberg et al, 2022; Hobohm et al, 2022; Voss et al, 2014; Kuhn et al, 2015). The reasons for this release are unclear, but it may serve to prevent the enzymes that have moved too far through the Golgi stack from accumulating on the cell surface in a membrane-bound form.

**Figure 1:**
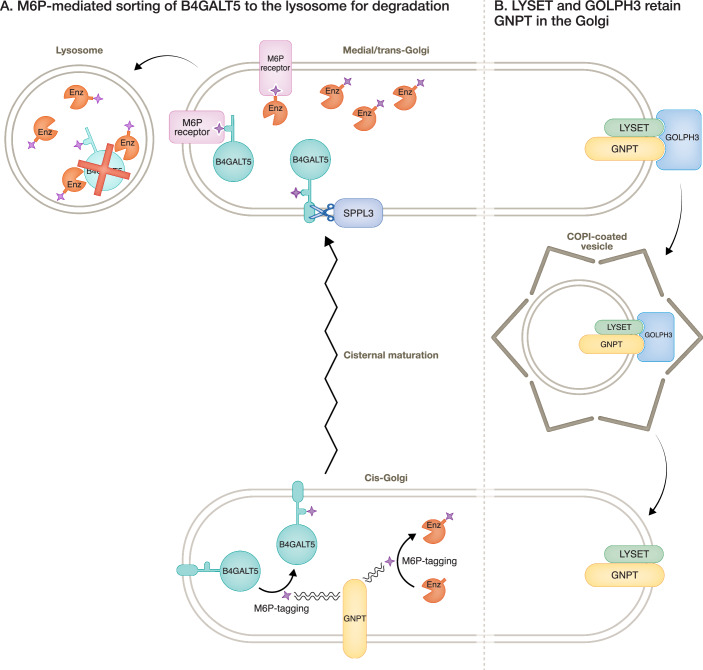
GNPT is retained in the Golgi to attach the M6P lysosomal sorting signal to lysosomal hydrolases and B4GALT5. (**A**) Just like the lysosomal hydrolases, the Golgi-resident glycosyltransferase B4GALT5 receives an M6P modification from the GNPT complex. The modified B4GALT5 is recognised by the M6P receptor in the trans-Golgi, directing its delivery to the lysosome for degradation. (**B**) The Golgi localisation of the GNPT complex is maintained by retrograde trafficking within COPI-coated vesicles. The sorting of GNPT into vesicles is mediated by binding to LYSET, which in turn binds the COPI coat via GOLPH3.

In a new study reported in this issue of the EMBO Journal, Brauer et al, reveal the mechanism of LYSET’s action in the M6P-tagging system and uncover an unexpected new role for M6P in the degradation of a Golgi-resident glycosyltransferase (Fig. 1). When the authors removed either LYSET or GNPT from cells they found that, as expected, many lysosomal enzymes were secreted as they were no longer tagged with M6P and so could not be recognised by the M6P receptors. However, the authors also noticed that loss of either LYSET or GNPT, or the chemical inhibition of M6P-addition, led to SPPL3-dependent hypersecretion of the Golgi-resident galactosyltransferase B4GALT5. Brauer et al, proceeded to show that B4GALT5 is subject to M6P modification and degradation; this led them to reason that, although M6P-tagging is typically thought of as a signal that targets lysosomal hydrolases to the lysosome, it can also target at least one Golgi protein for lysosomal degradation.

But how do LYSET and GNPT manage to navigate continuously to the correct position within the Golgi stack? The authors find that, for this, the COPI adaptors GOLPH3 and GOLPH3L are needed. LYSET acts as an essential bridge between GNPT (the cargo) and GOLPH3 (the COPI adaptor), indicating that the retention of some Golgi proteins might be more sophisticated than a simple ‘cargo-adaptor-coat’ arrangement. This provides one explanation for how hundreds of Golgi-resident proteins are retained by a single COPI vesicle coat using only the small number of ‘first order’ adaptors identified so far. In this way, ‘second order’ adaptors, such as LYSET in the case of GNPT, could be broadening the range of clients recognised by ‘first order’ adaptors such as GOLPH3. The authors’ in vivo data, together with AlphaFold 3 predictions, indicate a requirement for GOLPH3 to bind directly to LYSET to ensure the retention of GNPT in the Golgi. By establishing a precedent for such a retention system, this work opens the possibility that more ‘second order’ adaptors for specific cargos will be identified.

Overall, this study describes a single complex mechanism that both localises GNPT and degrades B4GALT5. At the same time, it highlights the importance of M6P-tagging machinery not only for the targeting of lysosomal enzymes, but also for the turnover of at least one (and maybe more) Golgi-resident proteins.
